# Salivary Scavenger and Agglutinin (SALSA) Is Expressed in Mucosal Epithelial Cells and Decreased in Bronchial Epithelium of Asthmatic Horses

**DOI:** 10.3389/fvets.2019.00418

**Published:** 2019-11-29

**Authors:** Gary Kwok Cheong Lee, Laurence Tessier, Dorothee Bienzle

**Affiliations:** Department of Pathobiology, University of Guelph, Guelph, ON, Canada

**Keywords:** asthma, equine, DMBT1, gp340, heaves, immunohistochemistry

## Abstract

The Salivary Scavenger and Agglutinin (SALSA) protein is an innate immune protein with various alleged functions, including the regulation of inflammation and tissue remodeling. Transcriptomic studies of severe equine asthma (SEA) showed downregulation of the gene encoding SALSA in bronchial epithelium of asthmatic compared to non-asthmatic horses. This study aimed to characterize expression of SALSA in equine tissues by immunohistochemistry (IHC), corroborate potential differences in epithelial gene expression between asthmatic and non-asthmatic horses, and assess the structure of equine SALSA. An antibody against SALSA was validated through immunoprecipitation followed by mass spectrometry and Western blotting to recognize the equine protein. This antibody was applied to tissue microarrays (TMAs) containing 22 tissues each from four horses. A quantitative PCR assay was designed to compare gene expression for SALSA between six asthmatic and six non-asthmatic horses, before and after an asthmatic challenge, using cDNA from endoscopic bronchial biopsies as source material. The *SALSA* gene from bronchial cDNA samples of 10 horses, was amplified and sequenced, and translated to characterize the protein structure. Immunostaining for SALSA was detected in the mucosal surfaces of the trachea, bronchi, bronchioles, stomach, small intestine and bladder, in pancreatic and salivary gland ducts, and in uterine gland epithelium. Staining was strongest in the duodenum, and the intercalated ducts and Demilune cells of the salivary gland. SALSA was concentrated in the apical regions of the epithelial cell cytoplasm, suggestive of a secreted protein. Gene expression was significantly lower (*p* = 0.031) in asthmatic compared to non-asthmatic horses. Equine SALSA consisted of three to five scavenger receptor cysteine-rich (SRCR) domains, two CUB (C1r/C1s, uegf, bmp-1) domains and one Zona Pellucida domain. These domains mediate the binding of ligands involved in innate immunity. Varying numbers of SRCR domains were identified in different horses, indicating different isoforms. In summary, equine SALSA has a predilection for mucosal sites, has multiple isoforms, and has decreased expression in asthmatic horses, suggesting alterations in innate immunity in equine asthma.

## Introduction

The Salivary Scavenger and Agglutinin (SALSA) is an innate immune protein with various putative functions, including regulation of inflammation, tissue remodeling and immune response (1, 2). The protein is known by numerous other names, including salivary agglutinin, glycoprotein-340, and Deleted in Malignant Brain Tumors 1 (DMBT1), but the name SALSA was most recently proposed to unify the original discovery in saliva with the ability to scavenge and agglutinate bacteria (3). The protein is extensively glycosylated and is secreted from mucosal surfaces. SALSA binds to a variety of endogenous and microbial ligands such as surfactant proteins A and D, complement proteins, and IgA (1, 4, 5). As such, SALSA interacts with components of both innate and adaptive immunity.

The ligand-binding property of SALSA resides in the nature and frequency of specific structural subunits: in humans, SALSA is comprised of three protein domains, namely the scavenger receptor cysteine-rich (SRCR) domain, the C1r/C1s, urchin embryonic growth factor and bone morphogenetic protein-1 (CUB) domain, and the zona pellucida (ZP) domain (6). The SRCR and CUB domains are involved in ligand binding, whilst the ZP domain appears to function in protein polymerization (6–8). The prototypic structure of human SALSA comprises a stretch of 13 SRCR domains, followed by two CUB domains on either side of a 14th SRCR domain, and a ZP domain at the C-terminal (1). However, this structure is not fixed, and SALSA isoforms of varying subunit composition and with a range of SRCR domains have been reported for individual persons (2, 9).

Because of mucosal localization and propensity to interact with various innate immune proteins, the role of SALSA has been investigated in conditions such as inflammatory bowel disease (IBD) and cystic fibrosis (CF), which are associated with altered mucosal barrier function, bacterial overgrowth, and cycles of inflammation (2, 10). Expression of SALSA at mucosal surfaces correlated positively with increasing degrees of inflammation, suggesting it was an induced protein and might have a protective role. This protective effect is believed to include negative feedback via innate inflammatory receptors such as Toll-like receptor (TLR) 4, thereby implicating SALSA as an immunomodulatory switch (2, 10). Conditions such as IBD and CF share some features with severe equine asthma (SEA), a common respiratory disorder also referred to as heaves or recurrent airway obstruction. Horses with SEA have severe neutrophilic small airway inflammation and alterations in the bronchial epithelium, and develop eventual lung remodeling and impaired gas exchange that becomes incompatible with life (11). Contrary to IBD and CF in humans, transcriptomic assessment of the equine bronchial epithelium identified downregulation of the gene encoding SALSA in asthmatic relative to non-asthmatic horses (11, 12). In light of these findings and the suggested roles of SALSA, we hypothesized that in horses, SALSA is concentrated at mucosal sites, has multifunctional domains, and is altered in inflammation. Here, we report on establishing an immunohistochemical assay for equine SALSA, evaluation of SALSA in multiple tissues, and gene expression in horses with and without asthma.

## Materials and Methods

### Antibody Validation

#### Western Blotting

A tissue lysate was prepared from equine duodenum collected immediately post-mortem. Duodenum was selected since SALSA is highly expressed in this tissue (2). Briefly, 50 mg of tissue were combined with 1 mL of lysis buffer (CelLytic^TM^ MT Cell Lysis Reagent; Sigma-Aldrich, St. Louis, MO, USA) and 10 μL of protease inhibitor cocktail (P8340; Sigma-Aldrich), and subjected to disruption in a TissueLyser II apparatus (Qiagen, Hilden, Germany). The lysed contents were centrifuged for 10 min at 4°C and 16,000 *g*. The supernatant was collected and represented the protein extract. Soluble proteins in the supernatant (30 μg) were separated by electrophoresis in an 8% SDS-polyacrylamide gel, and then transferred to a polyvinylidene fluoride (PVDF) membrane (Trans-Blot; Bio-Rad, Mississauga, ON, Canada). The membrane was washed in 1X wash buffer (Dako, Mississauga, ON, Canada) for 15 min, blocked with 3% skim milk for 1 h, and washed again with 1X wash buffer (Dako) for 15 min. The membrane was then incubated overnight at 4°C with anti-DMBT1 antibody (polyclonal rabbit IgG, concentration 1 μg/μL, RRID:AB_2818221, Sino Biological, Wayne, PA, USA) diluted 1:1,000 in 1% skim milk. The following day, the membrane was washed in 1X wash buffer for 45 min, with a change in buffer every 5 min, and then incubated for 30 min with secondary antibody [polyclonal goat anti-rabbit immunoglobulins-horse radish peroxidase (HRP); RRID:AB_2617138, Dako] diluted at 1:2,000 in 1% skim milk. Thereafter, the membrane was washed again over 45 min, and then incubated with enhanced chemiluminescence (ECL) Western blotting detection reagent (Clarity Western ECL; Bio-Rad). A Chemidoc^+^ instrument and ImageLab software (both Bio-Rad) were used to visualize and analyze, respectively, the membrane bands.

#### Immunoprecipitation

Supernatant (150 μg of protein extract) was incubated with 1 μL of a 1:100 DMBT1 antibody dilution on ice for 2 h, with manual rotation of the mixture every 15 min. Afterwards, 100 μL of protein A-coated magnetic microbeads (μMACS protein A microbeads; Miltenyi Biotec, Auburn, CA, USA) were added to the mixture, which was incubated on ice for 1 h. The mixture was passed through a μ column (Miltenyi Biotec) subjected to a magnetic field (μMACS separator; Miltenyi Biotec). The μ column was rinsed four times with 200 μL of lysis buffer (CelLytic^TM^ MT Cell Lysis Reagent; Sigma-Aldrich), four times with 100 μL of TrisHCl pH 7.5, and twice with 200 μL of 1X phosphate-buffered saline (PBS). The column was removed from the magnetic field, and 30 μL of PBS was passed through the column twice to dislodge the magnetic beads. The flow-through was collected and submitted for mass spectrometric analysis (LC-MS, SPARC BioCentre, The Hospital for Sick Children, Toronto, ON, Canada), along with a negative control sample prepared in an identical manner except for omission of antibody incubation. Mass spectra were analyzed using PEAKS studio (Bioinformatics Solutions, Waterloo, ON, Canada) and X! Tandem Alanine (The Global Proteome Machine, thegpm.org) software. PEAKS studio was set up to search the Uniprot-*Equus caballus*_Apr122019 database assuming digestion with trypsin. Tandems were searched with a fragment ion mass tolerance of 0.020 Da and a parent ion tolerance of 10.0 PPM. Carbamidoethyl was specified as a fixed modification. The bioinformatics tool Scaffold (version Scaffold_4.8.9, Proteome Software Inc., Portland, OR, USA) was used to validate the mass spectrometry results (13).

### Survey of SALSA Expression in Normal Tissues by Immunohistochemistry

Tissues collected from four horses donated and euthanized due to untreatable skeletal or orthopedic conditions were fixed in 10% neutral buffered formalin and embedded in paraffin. The horses were a 1 year-old male Thoroughbred with a deep tooth root abscess, a 1 year-old female Thoroughbred with osteochondritis of the right femur, a 6 year-old female Thoroughbred with degenerative disease in multiple joints, and a 17 year-old male Hannoverian with cervical arthritis. Tissue microarrays (TMAs) containing adrenal gland, bone marrow, cerebrum, esophagus, heart, kidney, large intestine, liver, lung, lymph node, pancreas, reproductive organ (testis or uterus), salivary gland, skin, small intestine (duodenum and jejunum/ileum), spleen, stomach, thyroid gland, tongue, trachea, and urinary bladder (22 tissues), were constructed from each horse using 1.0 mm tissue cores with 1.9 mm separations between each core. The TMAs were sectioned at 3 μm thickness with a rotary microtome and placed onto charged glass slides. Sections were de-paraffinized in xylene before immersion in pH 6.1 antigen retrieval solution (Dako) for antigen retrieval by heat (110°C for 1.5 min) in a de-cloaking chamber (Biocare Medical, Markham, ON, Canada). The slides were then washed in 1X wash buffer (Dako) for 5 min before a 10-min incubation step with dual enzyme blocker solution (Dako). Following another 5-min wash in 1X wash buffer, the slides were incubated with serum free protein blocker (Dako). The slides were subsequently incubated at room temperature for 2 h with primary anti-DMBT1 antibody (Sino Biological) diluted 1:750 in wash buffer. The slides were washed in 1X wash buffer for 45 min, changing the buffer every 5 min, before incubation at room temperature for 30 min with secondary antibody (polyclonal goat anti-rabbit immunoglobulins-HRP; Dako) diluted at 1:2,000. Nova Red chromogen (Dako) was applied as a chromogenic substrate for HRP, and the slides were counterstained for 3 min with Harris modified hematoxylin (ThermoFisher Scientific, Burlington, ON, Canada). Negative control slides were similarly processed, but with the omission of the primary antibody. The same immunohistochemistry (IHC) assay using anti-gp340 antibody (polyclonal rabbit IgG, concentration 10.5 mg/mL, diluted 1:100, and provided generously by U. Holmskov of Syddansk Universitet) as the primary antibody was also applied in parallel to tissues from the 1 year-old male Thoroughbred. Incubation with this primary antibody was overnight at 4°C, instead of 2 h at room temperature. Specificity of the antibody to equine SALSA was previously reported by U. Holmskov (4). Microscopic analysis was performed on a BX45 Olympus microscope, and images were acquired with a DP71 Olympus camera and cellSens standard 1.12 software. An IHC grading scheme was adapted to indicate the proportion of stained cells as 0 = none; 1 = <1%; 2 = 2–10%; 3 = 11–34%; 4 = 35–66%; or 5 = >66% (14, 15). Staining intensity was assessed semi-quantitatively as 0 = none; 1 = weak; 2 = moderate; 3 = strong. The score for proportion of stained cells was multiplied by the score for staining intensity, giving potential scores ranging from 0 to 15.

### Quantitative PCR

All procedures were approved by the University of Guelph Animal Care Committee (protocol R10 – 031) in accordance with the guidelines of the Canadian Council on Animal Care. Bronchial endoscopic biopsies were collected from six mature asthmatic (mean age of 15 years) and six mature non-asthmatic (mean age of 12 years) horses. The asthmatic horses had historically SEA diagnosed by clinical features including coughing and increased respiratory effort at rest, neutrophilic inflammation in bronchoalveolar lavage (BAL) fluid, abnormal respiratory function test results, and improvement in respiratory function following transfer to a low-dust environment. Duration of disease in asthmatic horses was 2–6 years, but they were free from clinical disease for at least 6 months prior to sample collection. The non-asthmatic horses had no historical or clinical evidence of respiratory disease, had normal respiratory function and normal BAL leukocyte populations. All horses were maintained outdoors for over 6 months prior to sample collection, before placement in a dust-free environment for 24 h. The horses were subsequently exposed to dusty hay for up to 3 days, or until the asthmatic horses showed respiratory impairment. Bronchial endoscopic biopsies were obtained from one lung before the dusty hay challenge, and from the contralateral lung after the challenge, resulting in a total of 24 samples, two from each horse. RNA was extracted from each endoscopic biopsy, and reverse transcribed into cDNA using a Superscript III Reverse Transcriptase kit (Invitrogen, Mississauga, ON, Canada) (11).

The cDNA was amplified in duplicate by qPCR using a LightCycler 480 instrument (Roche LifeScience, Laval, QC, Canada), SYBR green reagent (Roche LifeScience), and forward primer 5′-GCC CAC TGC TAC CCA AGA T-3′ and reverse primer 5′-TGA AGC CCA GGT TTA TGC GA-3′. The primers were predicted to be specific for *SALSA* mRNA (DMBT1, XM_014732986.1; predicted length 255 bp, GenBank) using the Basic Local Alignment Search Tool (BLAST) on the equine genome EquCab2.0 on the National Center for Biotechnology Information database (NCBI, Bethesda, MD). Reference genes were selected from a pool of five commonly used reference gene candidates that had been previously evaluated in equine samples: beta-actin (*BAC*), ribosomal protein L32 (*RPL32*), zeta polypeptide (*YWHAZ*), succinate dehydrogenase complex subunit A (*SDHA*), and glyceraldehyde-3-phosphate dehydrogenase (*GAPDH*) (16, 17). A preliminary qPCR run was performed with the candidate reference genes, and Normfinder software package was used to identify the two least variable genes (18). The two genes selected were *BAC* and *RPL32* as they were most stable and had similar cycle thresholds (Ct) to *SALSA*. Forward and reverse primers for *BAC* were 5′-GAC CCA GAT CAT GTT TGA GAC CT-3′ and 5′-TGA TGG AGT TGA AGG TAG TTT CGT G-3′, respectively. Forward and reverse primers for *RPL32* were 5′-GGG AGC AAT AAG AAA ACG AAG C-3′ and 5′-CTT GGA GGA GAC ATT GTG AGC-3′, respectively. As a calibrator, cDNA translated from RNA extracted from equine salivary gland tissue was used. The protocol included a 7-min pre-incubation phase at 95°C, 45 amplification cycles comprised of 20 s at 95°C, 20 s at 60°C, and 20 s at 72°C, a melting curve cycle comprised of 5 s at 95°C, 1 min at 45°C, and a continuous ramp rate of 0.11°C until 97°C, followed by a final 10 s cooling step at 40°C.

The qPCR efficiency for each gene tested was derived from standard curves. Relative gene expression was calculated using the equation:

$$\text{Relative}\;\text{gene}\;\text{expression}\;=\;\frac{{\left(E_{SALSA}\right)}^{\Delta Ct\;SALSA}}{GeoMean[{\left(E_{ref}\right)}^{\Delta Ct\;ref}]}$$

whereby “E” refers to the qPCR efficiency for the specified gene, “ΔCt” refers to the difference between the Ct of the calibrator and the specified gene, “ref” refers to the reference genes (repeated for each reference gene), and “GeoMean” refers to the geometric mean of gene expression of the two reference genes.

To assess the adequacy of the sample size in determining a significant difference in gene expression between asthmatic and non-asthmatic horses, the following equations were used (19):

$$\begin{array}{l} \;n_{A}=\;n_{B}\;=\;\left(1+\frac{1}{\kappa }\;\right){\left(\sigma \frac{z\left(1-\frac{\alpha }{2}\right)+z(1-\beta )}{\mu_{A}-\mu_{B}}\right)}^{2}\\ Statistical\;power=\Phi \left(z-z\left(1-\frac{\alpha }{2}\right)\right)+\Phi \left(-z-z\left(1-\frac{\alpha }{2}\right)\right)\;,\;\\ \;z=\;\frac{\mu_{A}-\mu_{B}}{\sigma \sqrt{\frac{1}{n_{A}}+\frac{1}{n_{B}}}} \end{array}$$

Here, A and B represent asthmatic and non-asthmatic horses, respectively, *n* is the sample size, κ is the matching ratio (i.e., 1 in this instance), μ is the mean, σ is the standard deviation, ϕ is the standard normal distribution function, α is Type I error, and β is Type II error, meaning 1 – β is the statistical power.

GraphPad Prism (Version 6.07 for Windows, La Jolla, CA, USA) was used for all subsequent statistical analyses. Relative gene expression results for each sample were log-transformed and tested for normality with a D'Agostino-Pearson test. An unpaired *t*-test with Welch's correction was used to compare relative gene expression between asthmatic and non-asthmatic horses. A ratio paired *t*-test was used to compare groups pre- and post-asthmatic challenge (asthmatic horses pre- and post-challenge, non-asthmatic horses pre- and post-challenge, and all horses pre- and post-challenge). A *p* < 0.05 was used as cutoff for statistical significance.

### Polymerase Chain Reaction for Whole Gene Sequencing

RNA extracted from bronchial endoscopic biopsies was reversed transcribed to cDNA using a Superscript III Reverse Transcriptase kit (Invitrogen). These endoscopic biopsies were the same ones described above. Six samples from non-asthmatic and three from asthmatic individuals yielded adequate cDNA following reverse transcription. An additional sample was obtained from bronchial mucosa collected from the 6 year-old female Thoroughbred with degenerative joint disease mentioned above. RNA was extracted from this sample and also reversed transcribed to cDNA, resulting in samples from 10 individuals. Primers for different amplifications were specific for the predicted *SALSA* mRNA (DMBT1: XM_014732986.1, DMBT1 protein-like: XM_023637966.1; GenBank, NCBI) and based on the equine genomes EquCab2.0 and EquCab3.0, respectively. Primer sequences are in Table 1, and were designed to amplify from nucleotides 102–3,026 on the predicted DMBT1 gene (XM_014732986.1), and from nucleotides 2,024–4,033 on the predicted DMBT1 protein-like gene (XM_023637966.1). The PCR amplifications were performed using a Platinum Taq DNA polymerase high fidelity PCR kit (Invitrogen). Each PCR included 5 μL of 10X high fidelity buffer, 1 μL dNTPs (0.04 mM), 2 μL MgSO_4_ (2 mM), 1 μL of each primer, 0.5 μL of Platinum Taq (2.5 U), and 1 μL of cDNA in 38.5 μL of water. PCR conditions for amplifications were 1 min at 94°C, followed by 35 cycles of 94°C for 30 s, annealing temperature as indicated in Table 1 for 30 s, and 68°C for 2 min, followed by a final elongation at 68°C for 10 min. The PCR products were loaded in a 1% agarose gel stained with SYBR Safe (Invitrogen) and separated at 120 V for 1 h. Amplicons of the expected size were cut out, followed by DNA extraction and purification (QIAquick; Qiagen, Toronto, ON, Canada). The purified DNA was submitted for automated sequencing (Laboratory Services Division, Guelph, ON, Canada) in forward and reverse direction. The sequences were assembled with the predicted *SALSA* mRNA sequences as templates (DMBT1: XM_014732986.1, DMBT1 protein-like: XM_023637966.1; GenBank, NCBI) using Geneious Pro software, version 11.0.2 (Biomatters, Auckland, New Zealand). Additional sequence alignments were generated with the following parameters: Global alignment with free end gaps, Gap open penalty 12, Gap extension penalty 3, cost matrix 65% similarity, and manual adjustment. Nucleotide sequences were translated into amino acid sequences, and conserved domains were identified using the NCBI Conserved Domain Database. Protein models were built using the T-cell differentiation antigen CD6 (UniProtKB- P30203) as template, and imported into the Swiss-PdbViewer software (SPDBV 4.1.0) for visualization. The isoelectric point for each protein was calculated in Geneious Pro software using the following parameters for amino acids: D = −3.9, E = −4.1, C = −8.5, Y = −10.1, H = 6.5, K = 10.8, and R = 12.5.

**Table 1:** Primers to amplify the equine SALSA gene for sequencing.

**Forward primer (5^**′**^-3^**′**^)**	**Reverse primer (5^**′**^-3^**′**^)**	**Annealing** **temperature (^**°**^C)**
GGAGACACAGACGCCAACT	TGGACCAGGTGTTGTGAGAAG	61
AGAGAAGATGCTGGAGTTGTG	GGATAGGACGGGCTGGAAAA	62
GCCTATGGTCTGCCTGTGAG	GAAGAGGTTTGAGCATCCGT	62
CAACGGATGCTCAAACCTCT	ACTCCAGCATCTTCGTGGTG	58
GCAACTGGGGGACAGTTTGT	GCTGGTCACACGATTGGAGA	60
ACACCTGGGTTGAGACGATG	AGGCCTGAGAAGCTGGTTTAT	59

## Results

### Antibody Validation

**Figure 1:**
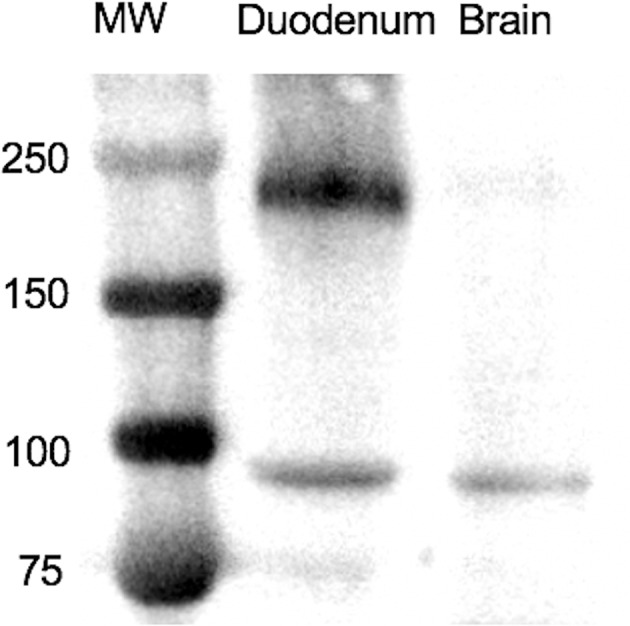
On Western blotting a protein of ~240 kD was recognized in a tissue lysate of duodenum. MW, molecular weight in kD.

A western blot was used to assess the specificity of the antibodies. The molecular weight of equine SALSA was estimated to be ~250 kD based on the translated nucleotide sequence and a prior report (20). Using the protocol described above, a prominent band of ~240 kD was identified (Figure 1). This band was very faint in a tissue lysate prepared from cerebrum of the same animal. Analysis of mass spectrometry data through Scaffold identified a peptide sequence (SGSSLSGSIK) within the immunoprecipitated protein extract that was absent in the sample prepared without primary antibody. This peptide sequence was predicted to be part of an ~232 kD uncharacterized protein and shared 100% identity with equine DMBT1 protein-like isoforms X1–X14 (XP_023493615.1, XP_023493622.1, XP_023493626.1, XP_023493634.1, XP_023493638.1, XP_023493647.1, XP_023493653.1, XP_023493660.1, XP_023493668.1, XP_023493675.1, XP_023493680.1, XP_023493689.1, XP_023493698.1, XP_023493705.1; GenBank, NCBI), and 80% identity with equine DMBT1 protein-like (XM_023637966.1; GenBank, NCBI). This peptide sequence had an observed mass to charge ratio (m/z) of 461.75, actual mass of 921.49 Da, a peptide charge of 2, a delta Da of 0.0089 and delta PPM of 9.624.

### Detection of SALSA in Normal Horse Tissues

**Figure 2:**
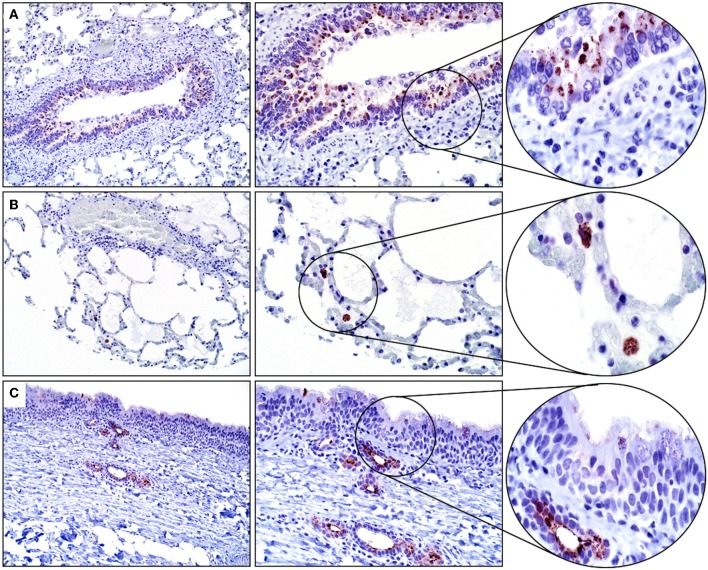
Immunohistochemical detection of SALSA in respiratory tissues. **(A)** Bronchiolar epithelium stains moderately intense in cytoplasmic, supranuclear, and apical locations. **(B)** Alveolar macrophages have intense granular cytoplasmic staining. **(C)** Tracheal mucosal epithelial cells have faint expression, while submucosal glands have moderate cytoplasmic staining; mostly along the luminal surface. Magnification ×200 (left), ×400 (middle), ×1,000 (right).

The results of IHC staining were similar for all four horses, and for both antibodies. Approximately 5% of tracheal mucosal, ciliated columnar epithelial cells had weak immunopositivity for SALSA. The staining location was cytoplasmic and supranuclear, and the pattern was granular, with positive staining concentrated along the luminal surface. Approximately 50% of goblet cells within the mucosal epithelium had cytoplasmic, granular immunopositivity for SALSA. Within the lower airways, most bronchial and bronchiolar ciliated columnar and goblet cells had moderate cytoplasmic, supranuclear, and apical granular immunopositivity for SALSA (Figure 2A). Approximately 5–10% of alveolar macrophages had intense cytoplasmic granular staining (Figure 2B). The majority of the epithelial cells of tracheal submucosal glands stained moderately positive for SALSA, with cytoplasmic granular staining, predominantly along the apical/luminal surface (Figure 2C).

**Figure 3:**
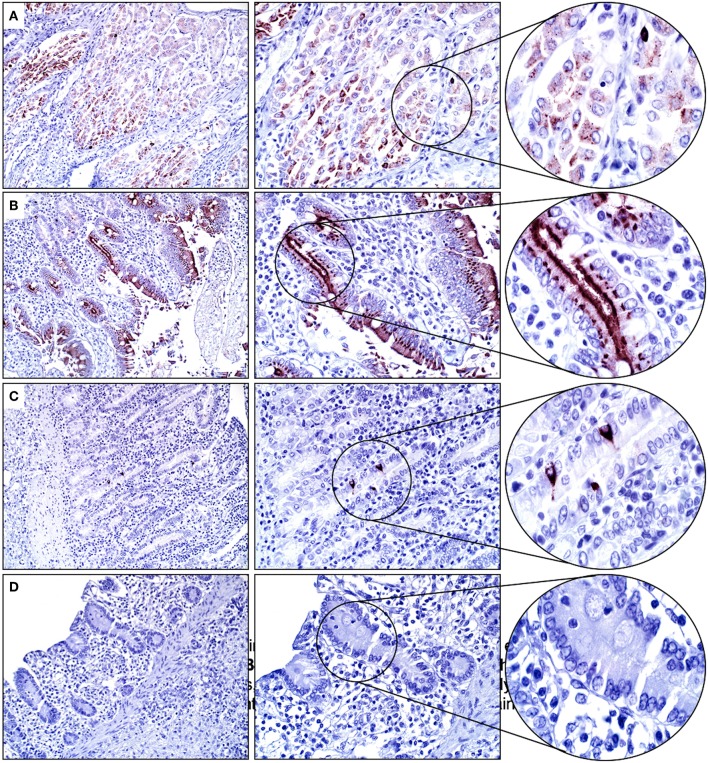
Glandular stomach and intestinal tract. **(A)** Gastric mucosal epithelial cells are moderately SALSA positive. **(B)** In the duodenum the surface epithelium, and in particular the apical aspect of cells, stains intensively positive. **(C)** Only rare individual epithelial cells in the distal small intestine have strong cytoplasmic staining. **(D)** No SALSA immunoreactivity is detected in the large intestine. Magnification as in Figure 2.

Mucosal epithelial cells of the glandular stomach, duodenum, and distal small intestines were positive for SALSA to varying degrees. Within the glandular stomach, most surface mucosal epithelial cells and submucosal mucous cells of the cardiac region had moderate granular and cytoplasmic, predominantly apical immunolabeling (Figure 3A). Duodenal mucosal epithelial cells (surface and crypt epithelial cells, including Paneth cells) had the strongest cytoplasmic immunopositivity for SALSA of all equine tissues. Most of these cells had strong supranuclear and apical immunolabeling (Figure 3B). In contrast, only rare mucosal epithelial cells within the crypts of the distal small intestines had strong granular, cytoplasmic and supranuclear immunopositivity for SALSA (Figure 3C). These cells could be columnar enterocytes, goblet cells, or enteroendocrine cells. The esophagus and large intestines were negative for SALSA (Figure 3D).

**Figure 4:**
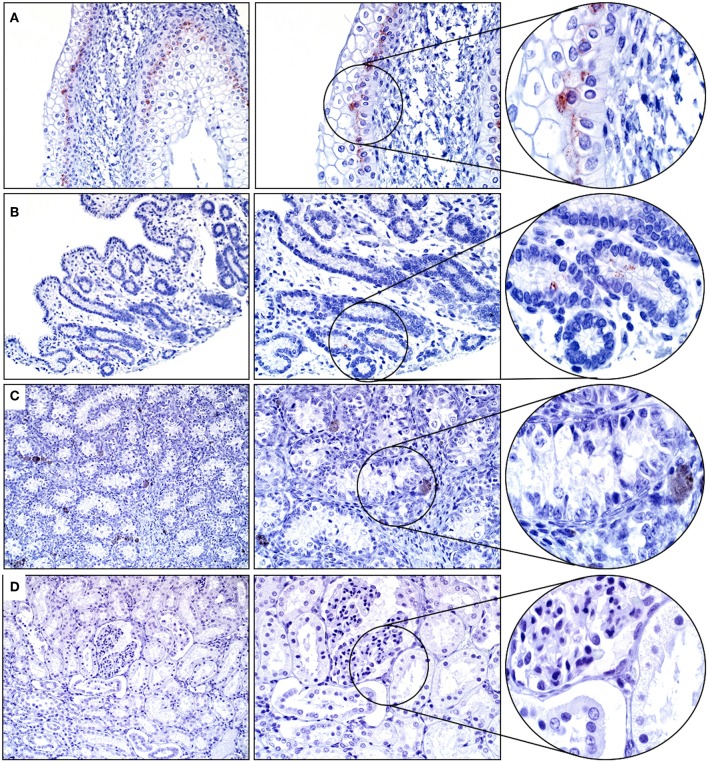
Genitourinary tract. **(A)** There is moderate SALSA immunoreactivity in the basal epithelial layer of the urinary bladder mucosa. **(B)** Only rare single uterine gland epithelial cells are positive for SALSA. **(C)** Testis and **(D)** kidney are negative for SALSA. There are a few pigmented interstitial cells in the testis that are a normal finding in young horses. Magnification as in Figure 2.

Approximately 60% of the cells within the basal layer of the urinary bladder's mucosa had immunopositivity for SALSA. The staining was cytoplasmic and supranuclear, and of moderate intensity (Figure 4A). Rare uterine gland columnar epithelial cells within the endometrium had moderate cytoplasmic and supranuclear immunopositivity for SALSA (Figure 4B). The kidneys and testes were negative on IHC for SALSA (Figures 4C,D).

**Figure 5:**
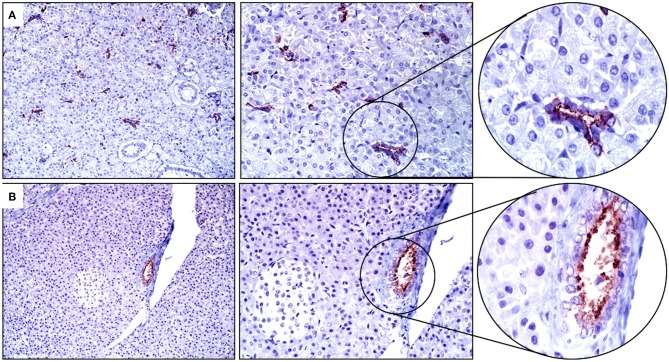
Salivary gland and pancreas. **(A)** There is strong cytoplasmic staining in intercalated duct and serous Demilune cells of the salivary gland but not in striated duct cells. **(B)** Pancreatic ducts have moderate staining concentrated along the apical surface. Neither sialocytes nor exocrine pancreatic cells are positive for SALSA. Magnification as in Figure 2.

Intercalated ducts and serous Demilune cells within the salivary gland had strong cytoplasmic and apical/luminal immunopositivity for SALSA (Figure 5A). Within the pancreas, epithelial cells of the intercalated and interlobular ducts had moderate cytoplasmic and apical/luminal immunolabeling for SALSA (Figure 5B).

Tissues from the adrenal gland, bone marrow, cerebrum, heart, liver, lymph node, spleen, skin, thyroid gland, and tongue were immunohistochemically negative for SALSA (Figure S1).

#### Summary of IHC

**Figure 6:**
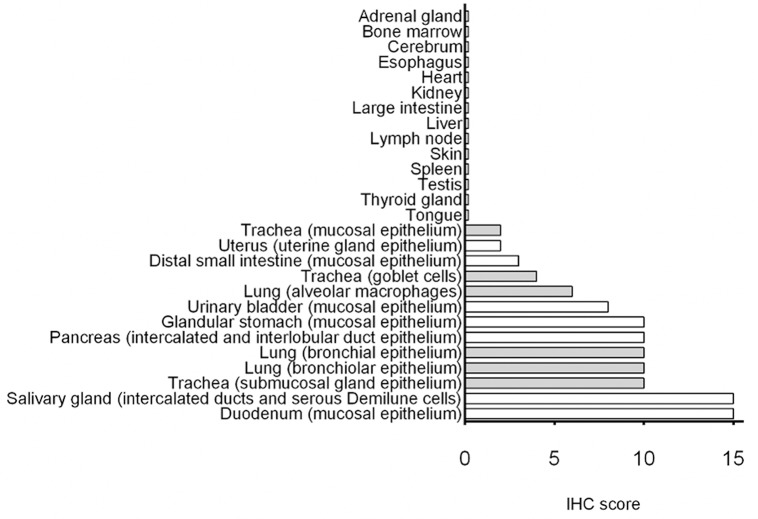
Summary of immunohistochemical detection of SALSA in 27 anatomical sites. IHC scores were calculated using a combination of staining intensity and proportion of cells with positive staining. Bars filled in gray represent tissues from the respiratory tract.

Cumulative IHC scores for the different organs are shown in Figure 6. Duodenum and salivary gland had the highest immunopositivity for SALSA. However, in salivary gland SALSA was not detected throughout the entire gland but was rather restricted to ductular structures. A similar but less intense pattern was noted in the pancreas. Detection of SALSA decreased distal to the duodenum, and was absent in the large intestines. There was overall moderate immunopositivity for SALSA throughout the larger airways, but not within the alveolar septa. Detection in the urinary bladder was moderate.

### Relative Gene Expression

**Figure 7:**
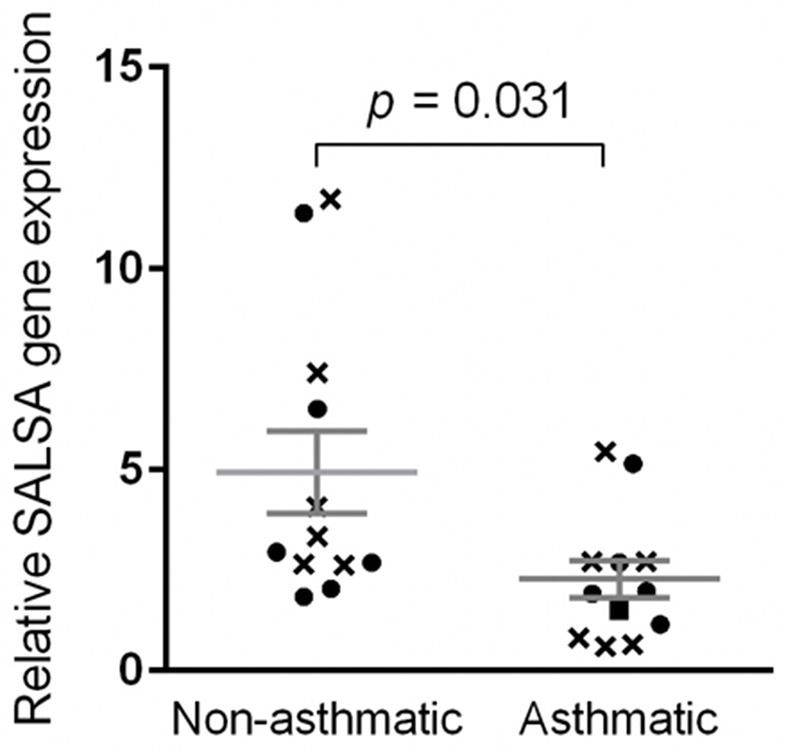
Relative *SALSA* gene expression was lower in asthmatic relative to non-asthmatic horses. Bar, standard error of the mean. Crosses and circles represent pre- and post-challenge samples, respectively.

The qPCR efficiencies for *BAC, RPL32*, and *SALSA* were 1.924, 1.967, and 1.916, respectively. Log-transformed data for relative gene expression passed the normality test (K2 = 0.008679, *p* = 0.9957). Non-asthmatic horses had mean relative *SALSA* gene expression of 4.94 ± 1.02 standard error of the mean (SEM) with a range of 1.84–11.73 and median of 3.14 (Figure 7). More specifically, non-asthmatic horses had mean pre-challenge gene expression of 5.31 ± 1.47 SEM with a range of 2.63–11.73 and median of 3.71, and mean post-challenge gene expression of 4.57 ± 1.53 SEM with a range of 1.84–11.37 and median of 2.81. Asthmatic horses had mean relative gene expression of 2.28 ± 0.46 SEM with a range of 0.61–5.45 and median of 1.95. More specifically, asthmatic horses had mean pre-challenge gene expression of 2.16 ± 0.77 SEM with a range of 0.61–5.45 and median of 0.82, and mean post-challenge gene expression of 2.40 ± 0.59 SEM with a range of 1.15–5.15 and median of 1.95. The standard deviations for all 24 samples, all 12 pre-challenge samples, and all 12 post-challenge samples were 3.01, 3.20, and 2.93, respectively.

Using the values obtained from all 24 samples, that is, a mean gene expression of 4.94 for non-asthmatic horses, a mean gene expression of 2.28 for asthmatic horses, a standard deviation of 3.01, and a type I error rate of 5%, the statistical power for the 24 samples was 0.87. When only the values for the 12 pre-challenge samples were used, the statistical power for these 12 samples was 0.68. Using the values for the 12 post-challenge samples, the statistical power was 0.44.

Comparing gene expression of all 12 samples from non-asthmatic horses to all 12 samples from asthmatic horses, the difference in gene expression between asthmatic and non-asthmatic horses was significant at *p* = 0.031. When only pre-challenge samples were included, the difference in gene expression between non-asthmatic and asthmatic horses was not significant (*p* = 0.097). There was also no statistically significant difference between the post-challenge samples of non-asthmatic and asthmatic horses (*p* = 0.2309). Differences in pre- compared to post-challenge samples of non-asthmatic (*p* = 0.3045, statistical power = 0.08) and asthmatic horses (*p* = 0.1715, statistical power = 0.07) were not statistically significant.

### Sequence and Structure of *SALSA*

**Figure 8:**
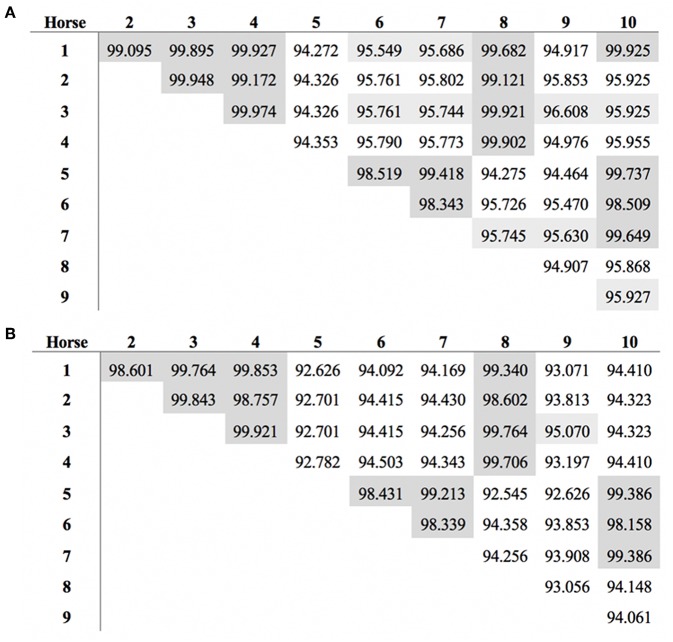
Similarity in *SALSA* **(A)** nucleotide and **(B)** amino acid sequence among 10 different horses. Shading indicates higher similarity.

**Figure 9:**
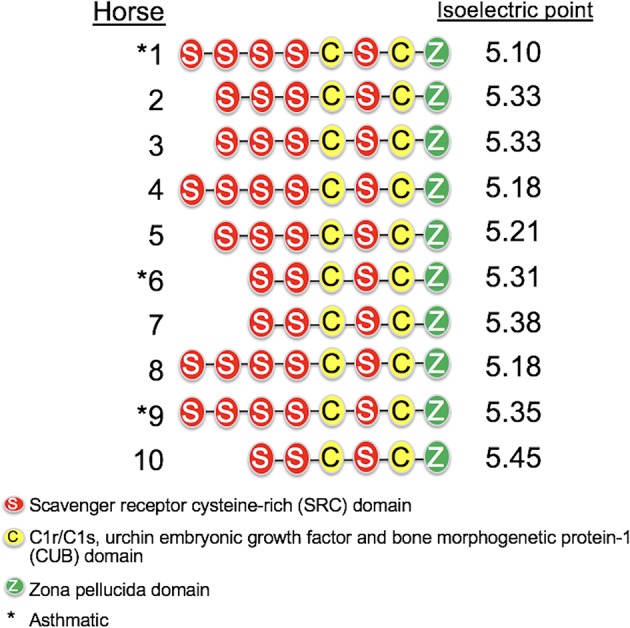
Protein structure of SALSA in 10 horses. The number of SRCR domains varied among horses, indicating different isoforms. The predicted isoelectric point also varied for different isoforms, and was generally higher for shorter isoforms.

**Figure 10:**
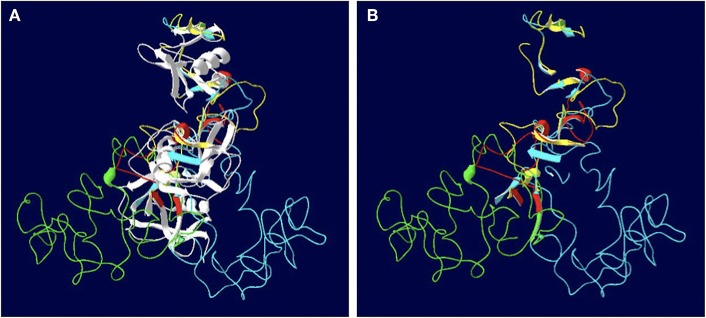
Models of the SALSA SRCR domains. **(A)** Superimposition of the SRCR domains from 10 individuals. Common strands are in white, and colored strands represent different isoforms. Most alpha helices and beta sheets are common to all 10 horses. **(B)** Same model with common strands omitted. Variable features between are largely due to protein loops rather than alpha helices and beta sheets.

The length of the *SALSA* nucleotide sequence obtained from 10 horses ranged from 3,421 to 4,274 bp. These sequences were submitted to GenBank (accession numbers MN065801, MN065802, MN065803, MN129174, MN129175, MN129176, MN129177, MN129178, MN129179, and MN129180). Among the 10 horses, nucleotide identity ranged from 94.3 to 99.9% and identity between the translated amino acid sequences ranged from 92.5 to 99.9% (Figure 8). The nucleotide sequences and their translated amino acid sequences shared 83.2–83.4% and 71.9–74.7% identity, respectively, with the human equivalent (DMBT1 isoform X1, *Homo sapiens*; XM_011539388.3, XP_011537690.1; GenBank, NCBI). Analysis of specific domains revealed a high degree of conservation of the SRCR, CUB, and ZP domains between horses and humans, and individual variation in the number of SRCR domains between different horses (Figure 9). All horses had two to four SRCR domains, followed by another SRCR domain sandwiched between a CUB domain on either side, and a ZP domain at the C-terminal. The majority of nucleotide and amino acid differences were within the SRCR domains, specifically in the region of nucleotide position 1–1,355 of the longest isoform. Within this region, in samples from horses 1, 2, 3, 4, 8, and 9 with the longest overall sequences, were multiple repeating stretches of nucleotides. Five of these sequences contained two identical 391-nucleotide stretches separated by eight identical nucleotides (GGACCGAG). The remaining sequence from horse 9, which was the longest sequence overall, had three similar 391-nucleotide repeats throughout the sequence, with each repeat separated by the same eight nucleotides. Although horse 5 had three consecutive SRCR domains (like horses 2 and 3), one of the 391-nucleotide repeat was not full length but lacked 15 nucleotides compared to those of other horses. The theoretical, calculated isoelectric points for the 10 proteins ranged from 5.10 to 5.45 (Figure 9). Modeling of SALSA SRCR domains revealed highly similar conserved alpha helices and beta sheets, and moderate variability in protein loops (Figure 10).

## Discussion

Although equine and human SALSA are highly similar, and the antibody employed was raised against full-length human DMBT1/SALSA, validation for use in a different species is essential (21). In this study, three approaches to antibody validation were employed: western blotting of a tissue with likely high (duodenum) and low (brain) expression, mass spectrometric identification of the appropriate target protein after immunoprecipitation, and analysis of results obtained with an antibody from a different source. Equine SALSA is not annotated in the UniProt database, therefore the peptide signature detected by mass spectrometry had to be correlated to the BLAST database. There was a slight discrepancy between the apparent mass spectrometry and immunoblotting results (232 kD vs. ~240 kD, respectively), which was likely attributable to only approximate size estimates with immunoblotting. Furthermore, positive and negative tissues were present in sections of each IHC assay, and omission of the primary antibody yielded no staining. Thus, the IHC assay was considered specific with a high degree of confidence.

In horses, immunohistochemically, SALSA was predominantly detected at mucosal sites, including the respiratory tract. The cytoplasmic, and typically supranuclear to apical, localization of the protein suggests luminal secretion into the airways and gastrointestinal tract. Location and putative secretion of SALSA in horses was similar as reported for humans (22). In addition to mucosal surfaces, SALSA was also identified in cells of the salivary gland responsible for serous secretions. Therefore, SALSA is located and likely secreted on surfaces that are typically exposed to foreign antigens, consistent with its putative role in innate immunity (2). A small proportion of alveolar macrophages was noted to be immunopositive for SALSA. Mucins, produced by the epithelium and submucosal glands of conducting and central airways, mixes with alveolar fluids, eliciting phagocytosis of mucins by alveolar macrophages (23). Mucins are expected to be rich in SALSA, consistent with epithelial cell immunopositivity, which would then explain presence of SALSA within some alveolar macrophages. Mucus and alveolar macrophages are both players in the innate immune defense of the airways. Within the gastrointestinal tract, expression of SALSA was pronounced within the stomach and duodenum, but decreased distally and became absent in the large intestine. Staining intensity varied slightly amongst submucosal mucous cells of the cardiac region, likely due to varying amounts of mucus within each cell. Within the distal small intestines, only rare epithelial cells within crypts were immunopositive for SALSA. Given the distribution of these cells, their possible identity might be enterocytes, goblet cells, or enteroendocrine cells. Immunopositivity for goblet cells would be consistent with the presence of SALSA in mucous secretions. On the other hand, immunopositivity in enteroendocrine cells is not expected given SALSA's limited role in hormone regulation, so these cells are less likely enteroendocrine cells. The large intestine is richer in commensal microflora than the more proximal intestinal segments, and resident microflora provides an important immunomodulatory environment, hence it is possible that the need for innate immune proteins such as SALSA is greater in the small intestine (24). Conversely, bacterial agglutination and inactivation via SALSA may be deleterious in the large intestine. The pancreas is connected to the duodenum by the pancreatic duct, therefore it might be beneficial for augmenting local innate immunity if pancreatic secretions that enter the duodenum are also rich in SALSA. Presence of SALSA within the epithelium of the urinary bladder also aligns with putative functions in innate immunity since the urinary bladder is a site that is susceptible to infections, especially ascending infections, and SALSA likely contributes to innate immune defense in that site. Within the bladder, SALSA was detected in the basal epithelial cells, but not in the superficial layers. The superficial bladder epithelium undergoes constant shedding with urination as a means to reduce bacterial load, whilst the basal cells are longer lived and serve as progenitor cells to replace the lost cells (25). Hence, it is possible that SALSA production is lost as a result of terminal differentiation in the superficial cells, or that it becomes redundant as these cells are shed.

Asthmatic horses had lower *SALSA* gene expression in bronchial epithelial biopsies than non-asthmatic horses. Differences in gene expression correlated with disease status rather than recent exacerbation. However, it should be noted that statistical power decreased below 0.80 whenever pre- or post-challenge samples were excluded from analysis, both a factor of smaller sample sizes and small differences between results. Inflammatory conditions such as bacterial pneumonia have been associated with increased expression of *SALSA*, presumably due to an ability to suppress the production of inflammatory cytokines, which differs from findings in this study (26–28). The expression of *SALSA* also increased upon respiratory bacterial infection in neonates, but a similar pattern was not noted in our horses following asthmatic challenge (27). Knowledge regarding *SALSA* expression in inflammatory disorders originates largely from studies in humans, and the function of SALSA may well be different in horses. Asthma has a particularly complex pathophysiology with strong environmental and genetic influences, which may uniquely alter *SALSA* expression. Severe asthma involves airway remodeling including smooth muscle hyperplasia, collagen deposition, and goblet cell and submucosal gland hyperplasia (29, 30). Goblet cell and submucosal gland hyperplasia are changes identified in asthmatic horses in remission, and absent in non-asthmatic horses (30). Both conditions involve cells that produce SALSA, which might contribute to the differential gene expression noted between asthmatic and non-asthmatic horses. However, SALSA was also present in ciliated bronchial epithelial cells, and the balance of contribution to gene expression by mucus-producing vs. ciliated cells under physiological and pathological conditions remains to be determined. Innate immune proteins are associated with alterations in the airway epithelium of human asthmatics, and mucus in human asthmatics differs in composition, viscoelastic properties and volume from that in non-asthmatics (12, 31). Therefore, it is likely that *SALSA* is not simply quantitatively related to production of mucus, but rather that the complex and chronic nature of SEA inflammation has diverse influences on gene expression. In addition, it was recently reported that greater concentrations of ambient fine particulate matter (PM_2.5_) were associated with lower levels of salivary SALSA in children (32). Fine particulate matter may downregulate innate immune proteins such as SALSA through a currently unknown mechanism, predisposing to respiratory infections (33). SEA can be induced by a variety of agents, including fine particulate matter (34). Therefore, prior exposure to particulate matter may also contribute to the lower gene expression of *SALSA* identified in asthmatic horses. Thus, it is likely that the expression of *SALSA* is influenced by multiple environmental and host conditions. Differences in expression between asthmatic and non-asthmatic horses may be more reflective of the underlying pathophysiology, which includes airway remodeling and environmental influences, rather than of inflammation alone or a specific disease entity.

Equine SALSA, like its human equivalent, is comprised of SRCR, CUB, and ZP domains. These domains mediate protein-protein interactions. For example, the SRCR domains bind to bacteria, and the CUB domains allow dimerization with complement components like C1q (35, 36). *In silico* analysis of human SALSA (UniProtKB- Q9UGM3) through FpClass, a data mining-based software used to predict protein-protein interactions, predicted interactions with innate proteins and receptors such as TLR4 (37). Inhibition of TLR4 signaling in response to lipopolysaccharide by recombinant SALSA has previously been demonstrated in human epithelial cell cultures, supporting an anti-inflammatory role (38). Similarity between equine and human SALSA, and the orthologs identified in other species, such as hensin in rabbits, CRP-ductin in mice, and ebnerin in rats, indicates strong evolutionary conservation (10, 39). Mice rendered genetically deficient in SALSA have increased expression of inflammatory cytokines such as TNF, IL6, and NOD, and humans naturally deficient in SALSA are at increased risk of developing Crohn's disease, further suggesting that SALSA functions to dampening inflammation (22). Given the inflammatory nature of SEA, it is hypothesized that SALSA ameliorates the disease, and that reduced expression of *SALSA* in asthmatic horses further accentuates the inflammatory process.

Variation in length of SALSA between horses is due to variations in the SRCR domains, as it is humans (2). The SRCR domains have a high degree of similarity, conveying susceptibility to alternative splicing and resulting in varying numbers of nucleotide repeats (2). Six of 10 horses had SRCR repeats (horses 1, 2, 3, 4, 8, and 9). In one instance, there were three repeats of the same 391-nucleotide sequence, resulting in a longer isoform. It has been hypothesized that shorter SALSA isoforms have reduced ability to agglutinate bacteria, thereby predisposing individuals to a pro-inflammatory response. As such, individuals with short isoforms were considered more prone to Crohn's disease and had reduced bacterial binding, but association with Crohn's disease was not universal (22, 35, 40). In this study, SRCR domain number appeared unrelated to asthmatic status, however, analysis of 10 individuals is insufficient to draw conclusions regarding disease association of copy number variants (CNV) in a population. It is also likely that there are additional isoforms not identified in this small sample of horses.

It should be noted that the primers used to sequence the *SALSA* gene begin at nucleotide 102 of the predicted equine *SALSA* gene (XM_014732986.1). Other primers were initially designed to amplify the region preceding nucleotide 102, but they instead amplified regions downstream of nucleotide 102. It may be that this region was not present in the specific horses, since there were segments within that predicted sequence that were not present in the final sequenced gene, or that the high degree of similarity between SRCR domains caused preferential amplification of downstream nucleotide regions. The predicted *SALSA* gene upon which primers were based was recently modified following an update to the equine genome (EquCab3.0, GCF_002863925.1). The revised predicted gene sequence (XM_023635156.1) is shorter (1,402 nucleotides) and only includes three SRCR domains. The current predicted gene is not annotated in databases other than GenBank, and the location within the equine genome is still unknown. The sequences described in this publication result from Sanger sequencing of two predicted mRNAs (XM_014732986.1 and XM_023637966.1) amplified with overlap and aligned, and therefore represent the most complete *SALSA* sequences in horses to date. As mentioned above, it is likely that these sequences will be updated further with discovery of additional isoforms.

Models of different SALSA isoforms revealed consistency in number and arrangement of alpha helices and beta sheets, but variability among the protein loops (Figure 10). Amino acid sequences within protein loops are typically relatively variable, even within the same protein family (41). While protein loops contribute little to protein stability, variations may alter protein shape and function (41). In the case of SALSA, since variations are largely due to variable number of repeats, binding affinity rather than overall function may be more likely affected. Protein modeling is least accurate when depicting protein loops; thus, reliability of models in determining function is limited (42).

The predicted isoelectric point between the different isoforms varied slightly. An acidic pH indicates that the protein is negatively charged at neutral pH. However, different anatomic locations vary in pH. For example, SALSA is present in both the stomach and duodenum, and these two sites are subject to vastly different pH environments. This suggests that SALSA binding affinity and function may also be site-dependent. Inflammation may also affect pH such as in humans with asthma who had airway acidification with a mean pH of 7.06 in uncontrolled asthmatics compared to a mean pH of 7.54 in healthy patients (43).

In summary, SALSA in horses is a multi-domain protein with a predilection for mucosal sites. Epithelial gene expression was lower in asthmatic compared to non-asthmatic horses, which may be related to airway remodeling, altered mucous secretion, prior exposure to particulate matter, and immune dysregulation. As in humans, repeats in the nucleotide sequence for SRCR domains result in different isoforms in horses. Future studies should focus on identifying potential SALSA functions in relation to inflammation and SEA.

## Data Availability Statement

The datasets generated for this study can be found in Genbank: https://www.ncbi.nlm.nih.gov/nuccore/. Accession numbers: MN065801, MN065802, MN065803, MN129174, MN129175, MN129176, MN129177, MN129178, MN129179, MN129180.

## Ethics Statement

The animal study was reviewed and approved by Animal Care Committee, University of Guelph.

## Author Contributions

GL designed and performed all parts of this study, excluding sequencing and mass spectrometry, and wrote the manuscript. LT contributed to the design of the study, PCR assays, and manually curated transcriptomic results for different SALSA isoforms. DB designed, funded, supervised all aspects of the study, and edited the manuscript. All authors have read and approved the manuscript.

### Conflict of Interest

The authors declare that the research was conducted in the absence of any commercial or financial relationships that could be construed as a potential conflict of interest.

## Supplementary Material

Figure S1 SALSA was not detected in **(A)** adrenal gland, **(B)** bone marrow, **(C)** cerebrum, **(D)** esophagus, **(E)** heart, **(F)** liver, **(G)** spleen, **(H)** skin, **(I)** thyroid gland, and **(J)** tongue. Magnification ×400.
