# Supplementary figures and images for: Salivary Scavenger and Agglutinin (SALSA) Is Expressed in Mucosal Epithelial Cells and Decreased in Bronchial Epithelium of Asthmatic Horses
Source: Front Vet Sci. 2019 Nov 29;6:418. doi: 10.3389/fvets.2019.00418 (PMC6896824; doi:10.3389/fvets.2019.00418)

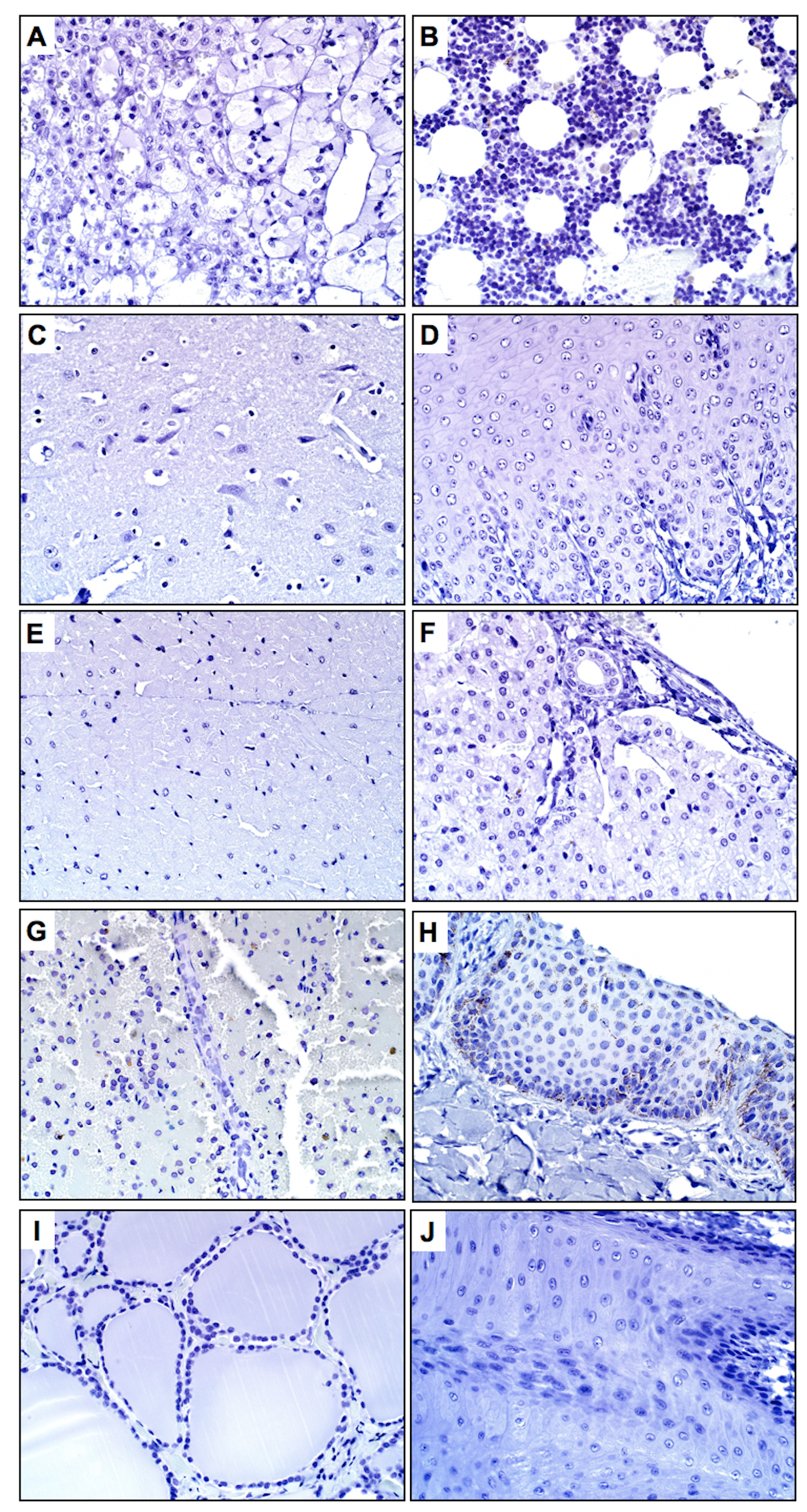

Supplement: Figure S1 — SALSA was not detected in (A) adrenal gland, (B) bone marrow, (C) cerebrum, (D) esophagus, (E) heart, (F) liver, (G) spleen, (H) skin, (I) thyroid gland, and (J) tongue. Magnification ×400. [file Image_1.TIFF]
